# P087: Multiresistant bacteria bacteremia cases in a Dakar University Hospital (Senegal)

**DOI:** 10.1186/2047-2994-2-S1-P87

**Published:** 2013-06-20

**Authors:** ML Dia, C Ndour, R Ka, R Diagne, A Diop, AI Sow, MF Cissé

**Affiliations:** 1 Laboratory of Bacteriology-Virology, CHU of Fann, Dakar, Senegal; 2 Infectious Diseases Department, CHU of Fann, Dakar, Senegal

## Introduction

The emergence of multiresistant bacteria strains compromises the efficiency of antibiotics usually used in our structures.

## Objectives

Our study had for aim to determine the part of multiresistant strains in bacteremia cases in the Teaching Hospital of Fann.

## Methods

This study was made on data recorded from registers of the bacteriological laboratory between 1 January 2008 and 31 December 2011.

## Results

One Hundred and forty six multiresistant bacteria (146) among the 709 multiresistant strains were isolated from blood cultures (20,59 %). The mean age was 27,18 years [range=1 – 84] with a sex ratio of 1.15. Most of the patients were hospitalized (91,1 %). The infectious diseases clinic provides most of the multiresistant bacteria (39, 72 %), followed by paediatrics department (37, 6 %) and Thoracic and cardiovascular Surgery department (6, 8 %). The majority of multiresistant bacteria was constituted by extended spectrum betalactamase enterobacteriaceae (82, 87 %) and Methicillin-resistant Staphylococcus (6, 85 %). Klebsiella pneumoniae was the most frequent bacteria (39, 72 %) followed by Enterobacter spp (23, 97). Enterobacteriaceae were susceptible to imipenem, amikacin and colistin but were resistant to quinolones and other aminosides. Methicillin-resistant Staphylococcus aureus and methicillin-resistant Staphylococcus saprophyticus were susceptible to vancomycin. Strains of Acinetobacter and Pseudomonas were susceptible to imipemem and colistin.

## Conclusion

Most of the multiresistant bacteria in the Teaching Hospital of Fann were isolated from blood cultures. It is important to insist on prevention by improving hospital hygiene and rational use of antibiotics.

## Disclosure of interest

None declared

